# Hyperthermia Induced by Gold Nanoparticles and Visible Light Photothermy Combined with Chemotherapy to Tackle Doxorubicin Sensitive and Resistant Colorectal Tumor 3D Spheroids

**DOI:** 10.3390/ijms21218017

**Published:** 2020-10-28

**Authors:** Catarina Roma-Rodrigues, Inês Pombo, Alexandra R. Fernandes, Pedro V. Baptista

**Affiliations:** UCIBIO, Department of Life Sciences, Faculdade de Ciências e Tecnologia, Universidade NOVA de Lisboa, 2829-516 Caparica, Portugal; catromar@fct.unl.pt (C.R.-R.); id.pombo@campus.fct.unl.pt (I.P.)

**Keywords:** 3D spheroids, photothermy, gold nanoparticles, doxorubicin resistance, colorectal cancer

## Abstract

Current cancer therapies are frequently ineffective and associated with severe side effects and with acquired cancer drug resistance. The development of effective therapies has been hampered by poor correlations between pre-clinical and clinical outcomes. Cancer cell-derived spheroids are three-dimensional (3D) structures that mimic layers of tumors in terms of oxygen and nutrient and drug resistance gradients. Gold nanoparticles (AuNP) are promising therapeutic agents which permit diminishing the emergence of secondary effects and increase therapeutic efficacy. In this work, 3D spheroids of Doxorubicin (Dox)-sensitive and -resistant colorectal carcinoma cell lines (HCT116 and HCT116-DoxR, respectively) were used to infer the potential of the combination of chemotherapy and Au-nanoparticle photothermy in the visible (green laser of 532 nm) to tackle drug resistance in cancer cells. Cell viability analysis of 3D tumor spheroids suggested that AuNPs induce cell death in the deeper layers of spheroids, further potentiated by laser irradiation. The penetration of Dox and earlier spheroid disaggregation is potentiated in combinatorial therapy with Dox, AuNP functionalized with polyethylene glycol (AuNP@PEG) and irradiation. The time point of Dox administration and irradiation showed to be important for spheroids destabilization. In HCT116-sensitive spheroids, pre-irradiation induced earlier disintegration of the 3D structure, while in HCT116 Dox-resistant spheroids, the loss of spheroid stability occurred almost instantly in post-irradiated spheroids, even with lower Dox concentrations. These results point towards the application of new strategies for cancer therapeutics, reducing side effects and resistance acquisition.

## 1. Introduction

Effective cancer eradication is being hampered by tumor cells’ acquired resistance to a wide spectrum of unrelated drugs, resulting in the so-called multidrug resistance (MDR) [1]. Despite the existence of different strategies for cancer treatment, including surgery, immune-, endocrine, gene, radio- or chemotherapies, the latter remains the method of choice [1]. However, exposure for a prolonged time to drugs, together with genetic alterations in tumor cells, hinder increased resistance to chemotherapeutics [1,2]. The acquired MDR, together with tumor heterogeneity, which is often correlated to characteristic tumor microenvironments, is forcing the development of new strategies to tackle cancer [1,3,4]. These strategies mainly rely on the application of combined therapies aiming to target multiple pathways in the tumor, circumventing MDR and lowering secondary effects [1,3,5,6].

Nanomedicine puts forward a multitude of conceptual tools to tackle cancer cells directly and/or to improve the delivery of therapeutic moieties, such as drugs, small interfering RNA (siRNA), antibodies, etc. [7,8,9,10,11]. Among these, nanoparticles able to convey different therapeutic cargos and with unique physico-chemical properties have allowed the design of novel combinatory strategies to tackle cancer [7,8,9,10,11]. Gold nanoparticles (AuNPs) possess remarkable optical properties that depend on their easy tunable size and shape and a large surface area to volume ratio, optimal for ease of functionalization with different agents [12]. Among these optical properties, the localized surface plasmon resonance (SPR) of AuNPs makes them suitable as photothermal agents in non-invasive photothermal therapy since they are extremely effective at converting light into heat [12,13,14,15,16,17,18,19,20]. Importantly, if the size is right, the irradiation may be in the visible region of the spectrum, allowing for visual control of the target subjected to therapy while improving the photothermal conversion ratio by using more energetic radiation than the conventional rear-infrared. What is more, this photothermy may be attained by means of green lasers commonly used in medical surgery for photocoagulation [16,21].

Thus far, most of the pre-clinical research and development of new cancer chemotherapeutics has been focused on two-dimensional (2D) cell cultures and murine animal models, whose limitations have been critical aspects for failure of clinical translation [22,23,24]. In fact, solid tumors are three-dimensional (3D) entities composed of tumor cells with variable proliferation, amidst oxygen and nutrient gradients, and communicating with a complex network of stromal, immune and endothelial cells in a characteristic extracellular matrix (ECM)—the tumor microenvironment [3,7]. Anticancer drugs are found in gradients in tumors, associated to deferential cell response. However, these intra-tumoral gradients are absent in 2D monolayers, which, coupled to differentially activated signaling pathways, might introduce a misleading bias when interpreting data originating from these in vitro 2D systems [25]. Another handicap of these 2D systems is the lack of ECM, which is preponderant for the mechanical properties of the tumor and improve intra- and inter-cellular communications [23]. Murine models, while substantially more complex to handle, still fall short of accurately representing the human tissues and organs [23]. To bridge this gap, 3D cultures, such as spheroids and organoids, have been introduced as relevant models for in vitro testing that combine the simplicity of 2D cell cultures with the three-dimensional, more complex tumor microenvironment (TME) [22,23]. Spheroids, consisting of 3D cell aggregates formed in ultra-low attachment coating microplates, mimic some important characteristics of tumors, such as the existence of ECM and the typical layering of solid tumors for spheroids with more than 200 µM [26,27,28,29]. The outermost proliferative zone with more oxygen supply and less interstitial pressure; the middle layer is the region with senescence cells (quiescent zone) characterized by higher interstitial pressure and middle level of oxygen supply; the innermost layer, or necrotic core, is characterized by low oxygen supply and high interstitial pressure with few to none cell proliferation [27,28]. The hypoxic and acidic environments found in the quiescent zone and necrotic core have been associated with low penetrability of drugs into tumors, and acquisition of radiotherapy and chemotherapy resistance [7,27,30,31]. The growth kinetics and the genetic expression of in vivo tumors are also mimicked in spheroids [26,27,28].

Herein, we evaluated the combination of photothermal irradiation using AuNPs with the chemotherapeutic agent Doxorubicin (Dox) to tackle a 3D model of colorectal cancer. Results suggested that spheroid treatment with AuNPs functionalized with polyethylene glycol (AuNP@PEG) combined with Dox and irradiation are promising therapeutic strategies to increase the therapeutic efficacy and decrease the dosage of the agent, even in drug-resistant cells.

## 2. Results and Discussion

We used 3D tumor spheroids to assess the drug gradient uptake and the effect of localized photothermy mediated by AuNP functionalized with polyethylene glycol (AuNP@PEG) alone or in combination with Dox. With that purpose, spheroids of colorectal cancer cells sensitive and resistant to Dox (HCT116 and HCT116-DoxR, respectively) were produced using ultra-low attachment plate wells. The HCT116-DoxR cell line was derived from a standard sensitive HCT116 cell line by culturing with increasing concentrations of Dox up to 3.6 μM [21].

### 2.1. Localized Irradiation of Spheroids

**Figure 1:**
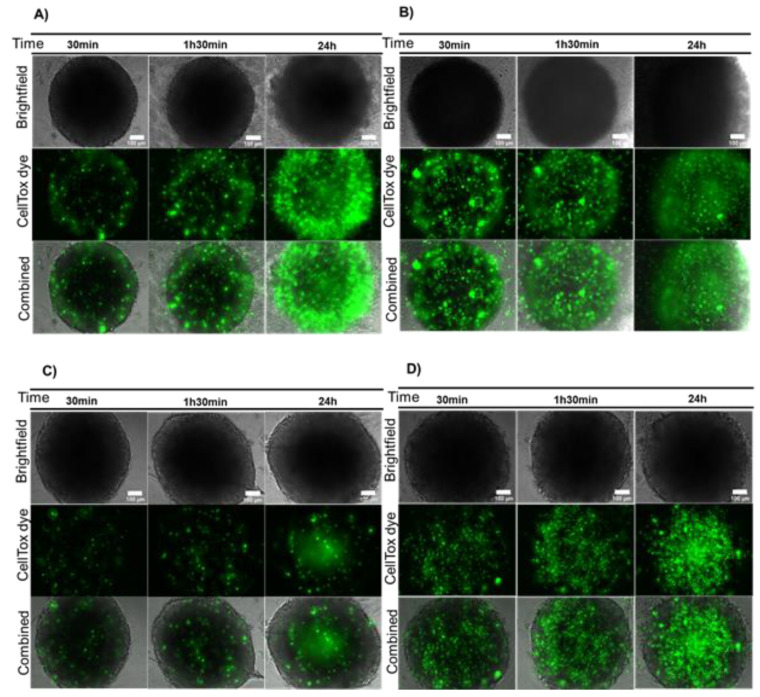
AuNP@PEG effect on cell viability measured by CellTox green dye. (**A**) HCT116 spheroids incubated for 24 h with 8 nM AuNP@PEG; (**B**) HCT116-DoxR spheroids incubated for 24 h with 8 nM AuNP@PEG; (**C**) HCT116 spheroids alone; (**D**) HCT116-DoxR spheroids alone. Microscopy images were acquired in Brightfield or with a green fluorescence filter to evaluate CellTox green dye fluorescence, after 30 min, 1 h 30 min or 24 h incubation. The combined images result from the overlap between Brightfield and green filter images. Scale bar corresponds to 100 μm.

Firstly, the spheroids’ cell viability was inferred after a 24 h incubation with AuNP@PEG, followed by 30 min, 1 h 30 min or 24 h incubation with a fresh medium supplemented with CellTox green dye. Results suggest a decreased viability of cells at the periphery of the spheroid after treatment with AuNP@PEG (Figure 1A,B) when compared to untreated spheroids (Figure 1C,D). The fluorescent peripherical corona increases over time, suggesting increased cell death induced by AuNP@PEG, which is corroborated by the disintegration of the spheroid observed in Brightfield images (Figure 1, Figure S1 and Movie S1–S4). A decrease in cell viability in the presence of AuNP@PEG has previously been observed and associated to reactive oxygen species (ROS)-mediated apoptosis [32,33,34,35]. After 24 h, there is an increase of fluorescence in the central area of the spheroids correlating to widespread cell death at the core (Figure S1). This effect might be associated to the diminished supply of nutrients and oxygen, indicating that a necrotic region was formed in HCT116 spheroids, mimicking those of tumors growing in vivo [28]. It is of particular relevance to observe the dynamics of fluorescence within the spheroid, which provides valuable information on the evolution of the overall systema rather than the momentary intensity of a particular observation field. The dynamics of fluorescence signal between core and periphery of the spheroid observed over time as function of challenging the structure with AuNP@PEG provide a more precise approximation to that of solid tumors (Figure S1).

**Figure 2:**
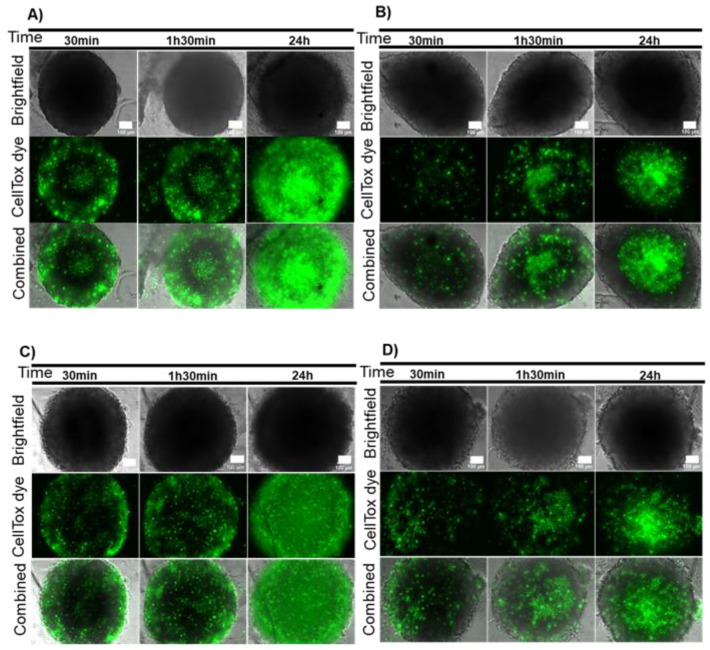
Combined effect of AuNP@PEG and irradiation on cell viability measured by CellTox green. (**A**) HCT116 spheroids incubated for 24 h with 8 nM AuNP@PEG and then irradiated with a 532-nm green laser for 1 min; (**B**) HCT116 spheroids irradiated with a 532-nm green laser for 1 min; (**C**) Doxorubicin-resistant HCT116 (HCT116-DoxR) spheroids incubated for 24 h with 8 nM AuNP@PEG and then irradiated with a 532-nm green laser for 1 min; (**D**) HCT116-DoxR spheroids irradiated with a 532-nm green laser for 1 min. Microscopy images were acquired in Brightfield or with a green fluorescence filter to evaluate CellTox green dye fluorescence, after 30 min, 1 h 30 min or 24 h incubation. The combined images result from the overlap between Brightfield and green filter images. Scale bar corresponds to 100 μm.

These spheroids were then subjected to localized photothermy mediated by AuNP@PEG [16,21]. HCT116 and HCT116-DoxR spheroids were incubated with AuNP@PEG as described above, washed three times to ensure removal of AuNP@PEG in suspension and irradiated with a 532 nm laser for 1 min, and cell viability was assessed with CellTox after 30 min, 1 h 30 min or 24 h. As observed for the AuNP@PEG alone, there is a more pronounced loss of viability of cells located at the periphery (Figure 2A–D). Analyzing this process with further detail shows that for HCT116 spheroids, the fluorescent signal increases over time, allowing the distinction of three layers (Figure 2A): (1) the peripheral layer that presents a high fluorescent signal that is not observed in irradiated spheroids (Figure 2B and Figure S2), suggesting that AuNP@PEG are responsible for cell death at the periphery; (2) a middle layer with low fluorescence; (3) the innermost core with increased fluorescence observed also in control spheroids (Figure 2B and Figure S2). Interestingly, HCT116-DoxR spheroids incubated for 24 h with AuNP@PEG and irradiated show scattered fluorescence throughout the spheroid, indicating extensive cell death culminating with the disintegration of the whole 3D structure (Figure 2C). The observed effect can be, at least partially, attributed to the photothermal effect, as previously reported [16]. In fact, the increase in temperature (ΔT) following 60 s irradiation is higher for spheroids pre-incubated with AuNP@PEG (ΔT = 10.2 °C), relative to irradiated non-treated spheroids (ΔT = 6.4 °C) or control, i.e., medium alone incubated with AuNP@PEG, washed three times with phosphate buffer saline (PBS) and then irradiated (ΔT = 6.6 °C).

When cells are irradiated without AuNPs, a pattern of cell death over time is also observed, with more fluorescent tumor cells in the inner core after 24 h (Figure 2B,D and Figure S2). This might be a side-effect of the light absorption by cytochromes present in cells, which are able to focus the irradiated light creating minute focal heating spots, but with considerably lower photothermal conversion efficiency when compared to AuNP@PEG. It should be noted that cells at the core of the spheroid are under stress due to limitations to the supply of nutrient and oxygen, which cumulatively contribute to the loss of viability in growing 3D cell structures [27,28,29]. The integrity of spheroids upon incubation with AuNP@PEG and irradiation was also followed in real time (Movie S5 and S6). Upon irradiation, HCT116 spheroids show a localized cell bursting effect at the periphery that follows the trend observed for AuNP@PEG gradient into the 3D structure. This localized photothermal effect continues for at least 48 h. Together, these results suggest that AuNP@PEG induce cell death in spheroid layers that is potentiated by irradiation.

### 2.2. Combination Therapy in 3D Models–Dox and Localized Photothermy Mediated by AuNP@PEG

Irradiation of AuNPs has been shown to promote cell permeabilization [18,19,20]. Hence, we hypothesized that AuNPs, with or without laser irradiation, might be able to increase drug penetration into spheroids, thus improving anti-tumor efficacy. A synergistic effect between photothermy mediated by AuNP@PEG and visible irradiation and Dox against breast cancer cells has been reported [16].

**Figure 3:**
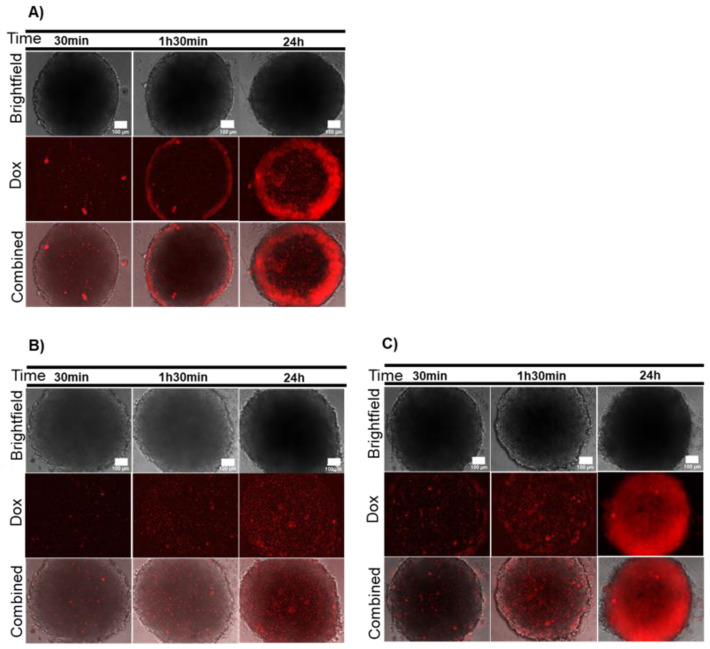
Dox diffusion in sensitive and resistant spheroids. (**A**) HCT116 spheroids incubated with 8 μM of Dox; (**B**) Dox-resistant HCT116 (HCT116-DoxR) spheroids incubated with 8 μM of Dox; (**C**) HCT116-DoxR spheroids incubated with 120 μM of Dox. Microscopy images were acquired in Brightfield or with a red fluorescence filter after 30 min, 1 h 30 min or 24 h incubation. The combined images result from the overlap between Brightfield and red filter images. Scale bar corresponds to 100 μm.

Firstly, we analyzed Dox diffusion in HCT116 and HCT116-DoxR spheroids via its intrinsic fluorescence (λ_exc_=470 nm; λ_em_=599 nm) [36]. Spheroids were incubated with 8 μM of Dox, corresponding to 20x the IC_50_ of Dox in a standard 2D monolayer of HCT116 cells (0.4 μM), and HCT116-DoxR spheroids were incubated also with an additional concentration of Dox (120 μM) [21,37] for 30 min, 1 h 30 min and 24 h (Figure 3). Dox showed a small diffusion into the central core of the spheroid and preferential accumulation at the periphery of HCT116 spheroids (Figure S3). In HCT116-DoxR spheroids, there was a low amount of fluorescence for the initial time points (30 min and 1 h 30 min) and equally distributed within the spheroid for both Dox concentrations (Figure S3). After 24 h of incubation with 120 μM of Dox, there was a noticeable increase in fluorescence at the periphery and intermediate areas of the spheroid, suggesting that Dox was accumulating in these sections (Figure 3C and Figure S3). In fact, previous reports have described a time-dependent penetration of Dox into HCT116 spheroids with the same pattern of distribution [38,39].

Cell viability evaluation via the CellTox green dye in these models seems to suggest that pre-incubation of sensitive and resistant HCT116 spheroids with Dox did not have an effect on the overall cell viability within the spheroids for the lower concentration (Figures S4 and S5).

We then followed the integrity of the spheroids in real time while monitoring the internalization of Dox by acquiring images every 15 min over a 48 h period. After 17 h incubation with Dox, disintegration of the 3D structure of HCT116 spheroid could be observed (Movie S7), as well as disintegration of HCT116-DoxR spheroid incubated with even lower concentrations of Dox after 32 h (Movie S8).

**Figure 4:**
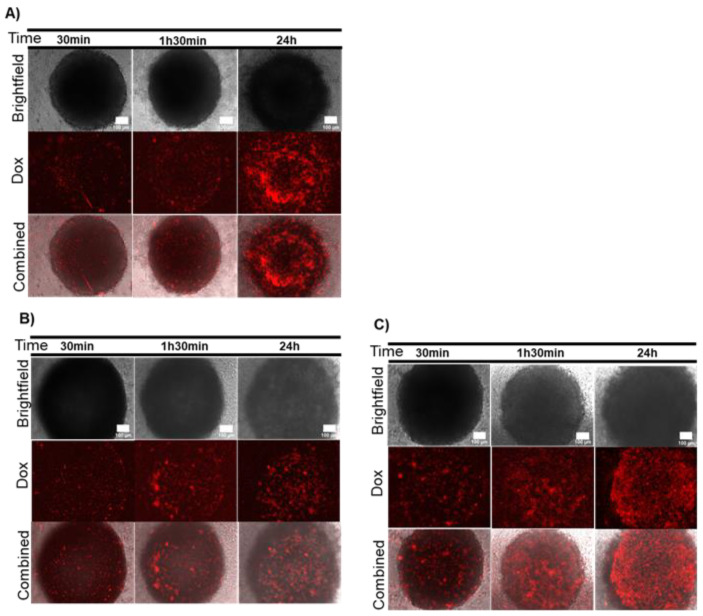
Doxorubicin (Dox) diffusion in spheroids after incubation with AuNP@PEG. (**A**) HCT116 spheroids incubated with 8 nM AuNP@PEG and then with 8 μM of Dox; (**B**) Dox-resistant HCT116 (HCT116-DoxR) spheroids incubated with 8 nM AuNP@PEG and then with 8 μM of Dox; (**C**) HCT116-DoxR spheroids incubated with 8 nM AuNP@PEG and then with 120 μM of Dox. Microscopy images were acquired in Brightfield or with a red fluorescence filter after 30 min, 1 h 30 min or 24 h incubation. The combined images result from the overlap between Brightfield and red filter images. Scale bar corresponds to 100 μm.

To assess the combinatory effect on 3D spheroids of chemo- and localized photothermy, we first pre-incubated the spheroids with AuNP@PEG and subsequently challenged these with Dox. As a result, an overall increase in red fluorescence in all spheroids was observed, suggesting that Dox had diffused faster and/or more easily within the 3D structures (Figure 4 and Figure S6). Curiously, there was a slight difference in observed accumulation of Dox between HCT116 and HCT1116-DoxR. The former showed higher accumulation of Dox in the intermediary region of the spheroid, whereas the latter showed a lower extent of penetration for the lower concentration of Dox (Figure 4 and Figure S6). Together, these data seem to suggest that the lower cell viability at the periphery (Figure 1) might allow deeper penetration of the drug into the spheroids due to disturbance of the 3D cell structure. This hypothesis is further supported by the observation that following pre-incubation with AuNP@PEG, the simultaneous incubation with Dox and the CellTox dye showed a general increase in green fluorescence at the intermediary section of these spheroids (Figure S7).

**Figure 5:**
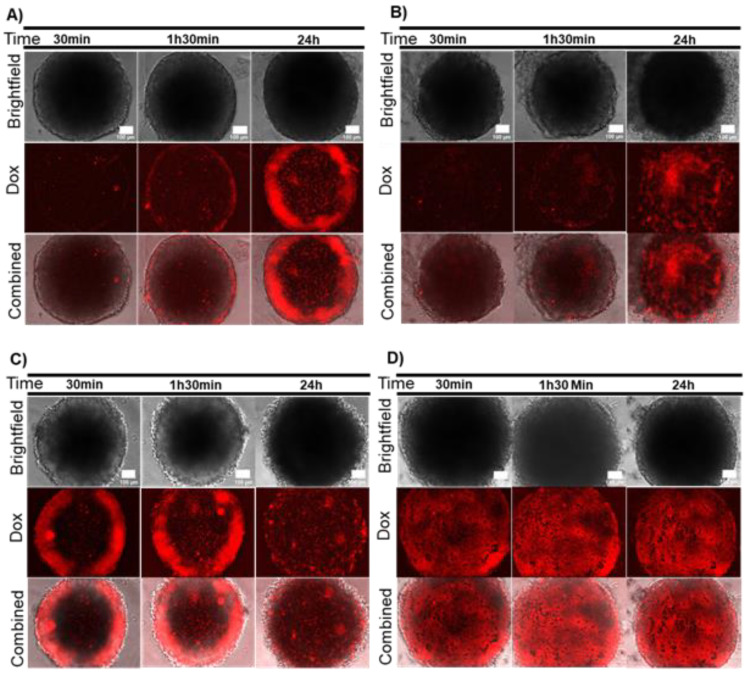
Effect of AuNPs and irradiation on Doxorubicin (Dox) diffusion in HCT116 spheroids. (**A**) HCT116 spheroids irradiated with a 532-nm laser for 1 min and then incubated with 8 μM Dox; (**B**) HCT116 spheroids incubated with 8 nM AuNP@PEG, irradiated with a 532-nm laser for 1 min and then incubated with 8 μM Dox; (**C**) HCT116 spheroids incubated for 6 h with 8 μM Dox and then irradiated with a 532-nm laser for 1 min; (**D**) HCT116 spheroids incubated with 8 nM AuNPs, incubated for 6 h with 8 μM Dox and then irradiated with a 532-nm laser for 1 min. Microscopy images were acquired in Brightfield or with a red fluorescence filter after 30 min, 1 h 30 min or 24 h incubation. The combined images result from the overlap between Brightfield and red filter images. Scale bar corresponds to 100 μm.

**Figure 6:**
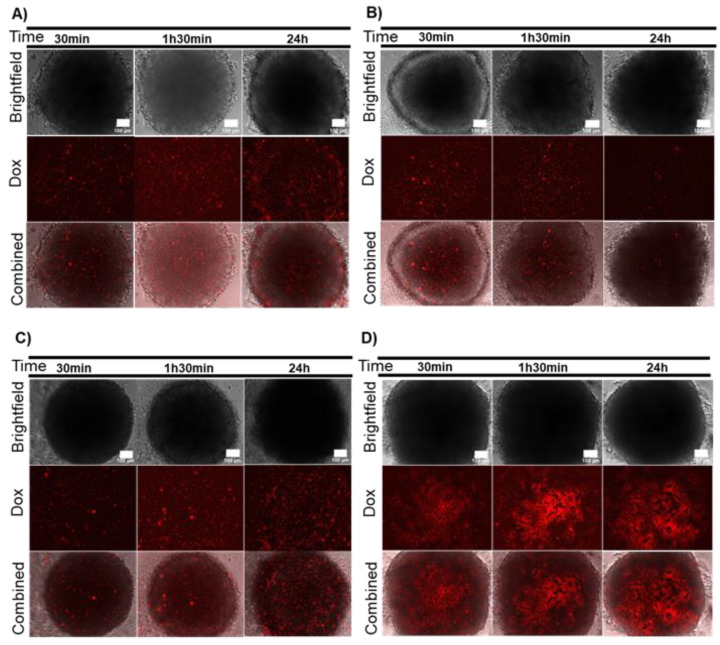
Effect of AuNPs and irradiation in Doxorubicin (Dox) diffusion in Dox-resistant HCT116 (HCT116-DoxR) spheroids. (**A**) HCT116-DoxR spheroids irradiated with a 532-nm laser for 1 min and then incubated with 8 μM Dox; (**B**) HCT116-DoxR spheroids incubated for 6 h with 8 μM Dox and then irradiated with a 532-nm laser for 1 min; (**C**) HCT116-DoxR spheroids incubated with 8 nM AuNP@PEG, irradiated with a 532-nm laser for 1 min and then incubated with 8 μM Dox; (**D**) HCT116-DoxR spheroids incubated with 8 nM AuNPs, incubated for 6 h with 8 μM Dox and then irradiated with a 532-nm laser for 1 min. Microscopy images were acquired in Brightfield or with a red fluorescence filter after 30 min, 1h 30 min or 24h incubation. The combined images result from the overlap between Brightfield and red filter images. Scale bar corresponds to 100 μm.

Figure 5 and Figure 6 show the effect of irradiation in the penetration of Dox into the spheroids. HCT116 spheroids incubated with Dox showed a similar penetration profile of the drug in pre-irradiated and non-irradiated cells (Figure 3A, Figure 5A and Figures S3 and S8). Noteworthily, spheroid disintegration occurs earlier for non-irradiated spheroids treated with Dox (after 17 h) while pre-irradiated spheroids treated with Dox disintegrate only after 24 h (Movie S7 and S9). Interestingly, irradiation after incubation with Dox resulted in the swelling of the spheroid with no visible disintegration until at least 48 h (Movie S10).

When HCT116 spheroids were pre-incubated with AuNP@PEG before irradiation and incubation with Dox, drug penetration seemed to be facilitated (Figure 5B). Although CellTox green staining does not seem to show increased cell death (Figure S9), the integrity of HCT116 spheroids incubated with AuNP@PEG, irradiated and then incubated with 8 μM Dox is highly affected, with total disintegration occurring after 12 h (Movie S11).

Irradiation of HCT116 spheroids after a 6-h incubation with Dox resulted in a distinct Dox accumulation profile when compared to pre-irradiated HCT116 spheroids and Dox incubation (Figure 5A,C). Figure 5C suggests that Dox accumulates at the peripheral layer of the spheroid at 30 min and then gradually diffuses through the whole 3D structure (at 24h). This effect is potentiated by the pre-incubation with AuNP@PEG before exposure to Dox and irradiation. Under these conditions, Dox is observed all over the HCT116 spheroid, suggesting high penetration of the drug in the first 30 min (Figure 5D). When assessing different time points for irradiation of spheroids incubated with AuNP@PEG, it becomes clear that the moment of irradiation is critical to maximize disintegration of the 3D structure (Movie S11 and S12). Only by observing the evolution of the 3D structure over time (i.e., movie vs. still image—see Supplementary Material), one can grasp the complexity of events taking place in these 3D models. If fact, the disintegration of the 3D structure leads to a bias in the quantification of fluorescence as cells shed from the spheroid, often taking the fluorophore with them (Figure S8D). Our data reinforce the importance of real-time imaging and monitoring of spheroid dynamics.

These results clearly indicate that combination of Dox and AuNP@PEG photothermal irradiation might be an excellent strategy to tackle 3D tumor structures. What is more, the harmonization of Dox accumulation and moment of irradiation is critical for spheroid destabilization, paving the way for the establishment of therapeutic strategies deferred in time to maximize efficacy.

Concerning the Dox-resistant spheroids (HCT116-DoxR), a similar penetration profile in pre-irradiated, post-irradiated and non-irradiated cells is observed (Figure 6A,B, Figure 3B and Figure S10), which is associated to lower cell death (Figure S11). The real-time analysis showed a similar behavior of pre-irradiated and non-irradiated spheroids incubated with 8 µM Dox, with disintegration observed after 30 h and 32 h, respectively (Movie S8 and S13). However, spheroid disintegration of post-irradiated spheroids occurs earlier—after 10 h of time of irradiation (Movie S14). Pre-incubation with AuNP@PEG showed an increase in drug diffusion within the spheroid, which is more evident when the spheroid is irradiated following incubation with AuNP@PEG and Dox (Figure 6C,D). Disintegration of the pre-irradiated HCT116-DoxR spheroid occurred at 30h, while that of the HCT116-DoxR spheroid incubated with AuNP@PEG, irradiated and incubated with Dox was observed after 6h (Movie S15). Once again, time of irradiation is critical to maximize disintegration and tumor ablation since irradiation after pre-incubation with AuNP@PEG and Dox resulted in an almost immediate loss of spheroid integrity (Movie S16). Once again, disintegration of the 3D structure leads to lower values of fluorescence intensity than expected (Figure S10D), pointing out the importance of real-time imaging and monitoring of spheroid dynamics.

An instantaneous increase to cell death was observed in the spheroid intermediary area when HCT116-DoxR spheroids were irradiated and then incubated with 120 μM Dox (Figure S12C). Once again, improved penetration of the drug occurs after pre-incubation with AuNP@PEG followed by irradiation and incubation with 120 μM of Dox, or vice-versa (Figure S12).

The cumulative effect of AuNP@PEG and irradiation might allow the use of lower doses of chemotherapeutic agents for the effective destabilization of tumor cells.

## 3. Materials and Methods

### 3.1. Gold Nanoparticles Synthesis, Functionalization and Characterization

AuNPs with a diameter of ~18 nm (18.4 ± 0.3 nm) were synthesized via the citrate methods and subsequently functionalized with polyethylene glycol (PEG, MW 350 g/mol, Sigma-Aldrich, St. Louis, MO, USA) to obtain 100% PEG coverage and characterized by dynamic light scattering for hydrodynamic size and zeta potential with a SZ-100 equipment from Horiba (Kyoto, Japan), and by transmission electron microscopy (TEM) in a JEOL 1200EX electron microscope (Tokyo, Japan) as previously described (Figure S13) [16,21,40].

### 3.2. Cell Cultures and Cell Cultures Maintenance

Colorectal carcinoma cell line HCT116 (CCL-247) was obtained from the American Type Culture Collection (ATCC^®^, Manassas, VA, USA). HCT116 Doxorubicin-resistant cell line (HCT116-DoxR) was derived from the sensitive HCT116 cells as previously described [21]. The HCT116 and HCT116-DoxR cultures were cultured as previously described [21]. Briefly, cells were maintained in Dulbecco’s Modified Eagle Medium (DMEM) (Thermo Fisher Scientific, Waltham, MA, USA) supplemented with 10 % (*v/v*) fetal bovine serum (FBS, Thermo Fisher Scientific, Waltham, MA, USA), and a mixture of Penicillin 100 U/mL and Streptomycin 100 μg/mL (Pen/Strep, Thermo Fisher Scientific, Waltham, MA, USA) at 37 °C with 99% (*v/v*) humidity and 5% (*v/v*) CO_2_. The culture medium for the HCT116-DoxR cell line was additionally supplemented with 3.6 µM Dox (for easier reading, supplemented media will be termed simply as DMEM).

### 3.3. Spheroids Preparation and Handling

HCT116 and HCT116-DoxR spheroids were prepared according to Baek et al. (2016), with few modifications [29]. Cultures were seeded at a density of 5 × 10^3^ cells per well in a super-low attachment 96-well culture plate (NunclonTM SpheraTM Microplate, Thermo Fisher Scientific, Waltham, MA, USA), shacked in an orbital direction and incubated for 7 days, when spheroids with a 750-µm diameter are obtained (Figure S14, HCT116, Movie S17, HCT116 and Movie S18, HCT116-DoxR).

After 7 days of growth, the medium was replaced by a fresh medium supplemented with 8 nM AuNP@PEG and incubated for 24 h. Spheroids were then washed three times for 1 min with PBS to remove non-internalized nanoparticles. HCT116 spheroids were incubated with DMEM (without phenol red) supplemented with 8 μM Dox (Merck, Darmstadt, Germany), and HCT116-DoxR spheroids were incubated with DMEM (without phenol red) supplemented with 8 μM or 120 μM Dox. As a control, spheroids were also incubated with Dox vehicle dimethyl sulfoxide (DMSO, Sigma-Aldrich, St. Louis, MO, USA), under the same conditions.

### 3.4. Spheroids Irradiation

The spheroids were irradiated with a 532- nm green diode-pumped solid-state laser (dpss) (Changchun New Industries Optoelectronics Tech. Co., Ltd., Changchun, China) coupled to a 1-mm diameter optical fiber with the tip placed 2 cm above the bottom of the well and a power set to 3.78 w.cm^−2^ for 1 min. The medium temperature before and after irradiation was measured using a multilogger thermometer hh806u (Omega Engineering, Norwalk, CT, USA).

### 3.5. Cell Viability

The CellTox^TM^ Green cytotoxicity assay (Promega Corporation, Madison, WI, USA) was used, following the manufacturer’s recommendations, for evaluating cell viability. Briefly, following spheroid incubation, the culture medium was removed and replaced by DMEM, without phenol red, supplemented with CellTox^TM^ Green dye 1x. The following settings were used: C1) spheroids incubated with CellTox green dye for 30 min, 1 h 30 min or 24 h; C2) spheroids were irradiated and then incubated with CellTox green dye for 30 min, 1 h 30 min or 24 h; C3) spheroids were incubated for 24 h with AuNP@PEG and then with CellTox green dye for 30 min, 1 h 30 min or 24 h; C4) spheroids were incubated for 24 h with AuNP@PEG, irradiated and then incubated with CellTox green dye for 30 min, 1 h 30 min or 24 h. Spheroids were observed and images were acquired with a Ti-U eclipse inverted microscope (Nikon, Tokyo, Japan) and respective software NIS Elements Basic software vs 3.1 (Nikon, Tokyo, Japan), using the Fluorescein isothiocyanate (FITC) filter (excitation at 480/30 nm and emission at 535/45 nm). The CellTox Green dye is a cyanine dye that enters cells with compromised membrane integrity and become fluorescent (excitation at 485–500 nm and emission at 520–530 nm) after binding to DNA, allowing the distinction between healthy cells (low fluorescence) from dead cells (high fluorescence). The cytotoxicity of the treatment could be inferred through the fluorescence intensity, as the fluorescent signal is proportional to the binding of the dye to the DNA of cells with compromised membranes [41,42]. ImageJ software (https://imagej.net/, version 1.53a) was used to measure the green fluorescence intensity (mean fluorescence x area) of the periphery and core (denser zone of the spheroid) of each spheroid. Bars in graphs represent the average of three different z-stacks of the peripheral and core zone of the spheroid (corresponding to middle layer and innermost core).

### 3.6. Effect of Doxorubicin

For the analysis of Dox diffusion, the following settings were used: D1) spheroids were incubated with DMSO for 30 min, 1 h 30 min or 24 h; D2) spheroids were incubated with Dox for 30 min, 1 h 30 min or 24 h; D3) spheroids incubated with Dox for 6 h, irradiated and incubated for 30 min, 1 h 30 min or 24 h; D4) spheroids incubated first with AuNP@PEG for 24 h, and then with Dox for 30 min, 1 h 30 min or 24 h; D5) spheroids incubated first with AuNP@PEG for 24 h, then with Dox for 6 h, irradiated and incubated for 30 min, 1 h 30 min or 24 h. Images of Dox diffusion were acquired in a Ti-U eclipse inverted microscope (Nikon, Tokyo, Japan) and respective software NIS Elements Basic software vs 3.1 (Nikon, Tokyo, Japan), using a red filter (G2A filter—excitation at 535/50 nm and emission > 580 nm). ImageJ software (https://imagej.net/, vs 1.53a) was used to measure the red fluorescence intensity (mean fluorescence x area) of the periphery and core (denser zone of the spheroid) of each spheroid. Bars in graphs represent the average of three different z-stacks of the peripheral and core zone of the spheroid.

The analysis of cell viability was also evaluated via incubation with CellTox Green dye after spheroids’ treatment (as described above).

Spheroids’ morphology was visualized via a cell culture video monitoring Cytosmart Lux2 (Cytosmart technologies, Eindhoven, The Netherlands) with Brightfield with digital contrast phase. The monitorization of Dox internalization in spheroids was performed for 2 days, with the exact time stated in movie caption, in a Fluorescent Lux2 (Cytosmart technologies, Eindhoven, The Netherlands), and red fluorescence with maximum exposure and gain 4. The focus of the microscope was set to be at the bottom of the spheroid. The loss of spheroid integrity was considered to begin when cell debris and/or many cells started to detach from the surface of the spheroid.

## 4. Conclusions

The combinatory effect of different therapeutic approaches has been put forward as an effective strategy to tackle drug resistance in cancer. Here, we have demonstrated the combinatory effect of Dox and localized photothermy mediated by spherical AuNPs against colorectal tumor cells in 3D spheroid models. Using these spheroid models, we highlight the effect of diffusion through the 3D structures in cell viability and structural organization of the tumor cells. Data suggest that AuNP@PEG are effective against cancer cells and induce severe loss of integrity in HCT116 and HCT116-DoxR spheroids. This is more so when combined with photo-induced hyperthermia. A combination of these approaches enhanced the penetration of the chemotherapeutic agent into the 3D structures. The moment of irradiation is critical to potentiate the loss of spheroid stability. These results seem to indicate that such a combinatory strategy may reduce the amount of drug needed to attain the same efficacy, thus reducing the possibility of side effects. In cancer cells already showing resistance to chemotherapy, this combinatory strategy may allow to increase the stress imposed to these cells and tackle resistance and relapse.

Our study clearly emphasizes the importance of real-time assessment of spheroid dynamics over time. Often, the effect of agents on spheroids is reported by evaluation of single time points, i.e., data retrieved from a still image. More important than the fluorescence intensity, per se, is the dynamic of fluorescence variation, both in time and space, namely between the core and the periphery of the spheroid. This is of particular relevance when combinatory strategies are being studied and evaluated, since each one of these ought to tackle a distinct pathway and/or chain of events, leading to ablation of the tumor. What is more, only dynamic acquisition of data allows to follow the structural alteration of the 3D spheroids (e.g., cell death and consequent disintegration) and the stages leading to this ultimate fate. Further studies will be needed to demonstrate whether this strategy might be broadly applicable to other chemotherapeutics used in clinical oncology.

## Supplementary Materials

Supplementary materials can be found at https://www.mdpi.com/1422-0067/21/21/8017/s1.
