# Supplementary material for: Hyperthermia Induced by Gold Nanoparticles and Visible Light Photothermy Combined with Chemotherapy to Tackle Doxorubicin Sensitive and Resistant Colorectal Tumor 3D Spheroids
Source: Int J Mol Sci. 2020 Oct 28;21(21):8017. doi: 10.3390/ijms21218017 (PMC7672550; doi:10.3390/ijms21218017)
Supplement: Supplementary file 1 [file ijms-21-08017-s001.zip › Roma-Rodrigues_SI/Supplementary materials_Roma-Rodrigues_2020_.pdf]

International Journal of *Molecular Sciences*

MDPI

Hyperthermia Induced by Gold Nanoparticles and Visible Light Photothermy Combined with Chemotherapy to Tackle Doxorubicin Sensitive and Resistant Colorectal Tumor 3D Spheroids

Catarina Roma-Rodrigues [†], Inês Pombo [†], Alexandra R. Fernandes * and Pedro V. Baptista *

UCIBIO, Department of Life Sciences, Faculdade de Ciências e Tecnologia, Universidade NOVA de Lisboa, 2829-516 Caparica, Portugal; catromar@fct.unl.pt (C.R.-R.); id.pombo@campus.fct.unl.pt (I.P.)

* Correspondence: ma.fernandes@fct.unl.pt (A.R.F.); pmvb@fct.unl.pt (P.V.B.); Tel.: +351-21-2948530 (P.V.B.)

† These authors contributed equally to this work.

Supplementary material

Movie captions:

Movie S1: Time course of HCT116 spheroid after 24h incubation with 8 nM AuNP@PEG. Spheroids were washed three times with phosphate buffer saline and incubated in fresh medium. Images were acquired every 15 min with contrast phase using Lux2 (Cytosmart) for 23h45min. The structure of the right side of the spheroid seems to be compromised as of the beginning of the experiment, with increased cellular detachment along time. Cell debris may be perceived since the beginning of the movie.

Movie S2: Time course of HCT116 resistant to doxorubicin spheroids (HCT116-DoxR) after 24h incubation with 8 nM AuNPs. Spheroids were washed three times with phosphate buffer saline and incubated in fresh medium. Images were acquired every 15 min with contrast phase using Lux2 (Cytosmart) for 1day 22h 50min. The integrity of the spheroid wall seems to be compromised as of the experiment. After 10h incubation, an increase of cell detachment from the 3D structure is observed.

Movie S3: Time course of the aspect of untreated HCT116 spheroid. Images were acquired every 15 min with contrast phase using Lux2 (Cytosmart) for 2 days. The spheroid membrane integrity is maintained throughout the experiment timeframe with few cells detaching from the structure.

Movie S4: Time course of the aspect of untreated HCT116 resistant to doxorubicin spheroids (HCT116-DoxR). Images were acquired every 15 min with contrast phase using Lux2 (Cytosmart) for 2 days. The spheroid structure remains cohesive throughout time, although some cells keep detaching from the surface and few debris may be observed.

Movie S5: Time course of the aspect of HCT116 spheroid incubated 24h with 8 nM AuNPs, washed three times with phosphate buffer saline and irradiated for 1 min with 532 nm laser. Images were acquired every 30 min with contrast phase and 20 x amplification using Lux2 (Cytosmart) for 48h. The integrity of the spheroid membrane seems to be maintained throughout the experiment, with bubbles, consistent with cells burst, continually appearing. After 15h, cell debris are observed at the top right of the spheroid.

Movie S6: Time course of HCT116 resistant to doxorubicin spheroids (HCT116-DoxR), incubated 24h with 8 nM AuNPs, washed three times with phosphate buffer saline, and irradiated for 1 min with 532 nm laser. Images were acquired every 15 min with contrast phase using Lux2 (Cytosmart) for 1 day 23h 15min. The spheroid membrane integrity throughout time seems to be identical to that of untreated spheroid (Movie S4), with increased formation of cell debris and cell detachment on the right upper side of the spheroid after 1day 11h 15 min.

Movie S7: Time course of the aspect of HCT116 spheroid incubated with 8 μM doxorubicin. Images were acquired every 15 min with contrast phase and red fluorescence using Lux2 (Cytosmart) for 1day 21h 47min. An increased Dox intensity is observed after 2h incubation, with complete spheroid coverage after 15h. The disintegration of the spheroid is observed after 17h incubation. A radical release of cells and debris is observed.

Movie S8: Time course of the aspect of spheroids of HCT116 resistant to doxorubicin (HCT116-DoxR), incubated with 8 μM doxorubicin. Images were acquired every 15 min with contrast phase and red fluorescence using Lux2 (Cytosmart) for 1day 21h 57min. Doxorubicin penetration in the spheroid is visualized after 4h incubation covering the entire spheroid after 21h. The spheroid size seems to increase after 12h incubation with increased and evident cell detachment after 32h.

Movie S9: Time course of the aspect of HCT116 spheroid irradiated for 1 min with 532 nm laser and then incubated with 8 μM doxorubicin. Images were acquired every 15 min with contrast phase and red fluorescence using Lux2 (Cytosmart) for 2days 0h 34min. The red fluorescence is observed to completely cover the spheroid after 13h. An increased accumulation of cell debris is observed after 24h.

Movie S10: Time course of the aspect of HCT116 spheroid incubated with 8 μM doxorubicin and then irradiated for 1 min with 532 nm laser. Images were acquired every 15 min with contrast phase using Lux2 (Cytosmart) for 2days 0h 34 min. After irradiation, the spheroid size seems to increase during the first 22h with cell bursting appearing on the spheroid exterior side. Although cell debris are observed after the first 5h after irradiation, no visible disintegration of the spheroid is observed until at least 2days.

Movie S11: Time course of the aspect of HCT116 spheroid incubated with 8 nM AuNP@PEG, washed three times with phosphate buffer saline, irradiated for 1 min with 532 nm laser in fresh medium and then incubated with 8 μM doxorubicin. Images were acquired every 15 min with contrast phase and red fluorescence using Lux2 (Cytosmart) for 1day 23h 46min. Doxorubicin total spheroid coverage is observed after 21h incubation. The disintegration of the spheroid, suggested by cell detachment and accumulation of cell debris, start after 12h with total loss of integrity after 1day 15h.

Movie S12: Time course of the aspect of HCT116 spheroid incubated with 8 nM AuNP@PEG, washed three times with phosphate buffer saline, followed by 6h incubation with 8 μM doxorubicin and irradiation for 1 min with 532 nm laser. Images were acquired every 15 min with contrast phase using Lux2 (Cytosmart) for 1day 18h 30min. The spheroid size increases during the first 17h with occasional cell bursting. After 24h, the disintegration of the spheroid is observed, with an increased accumulation of cell debris and the detachment of cells.

International Journal of *Molecular Sciences*

Movie S13: Time course of the aspect of spheroids of HCT116 resistant to doxorubicin (HCT116-DoxR), irradiated for 1 min with 532 nm laser and then incubated with 8 μM doxorubicin. Images were acquired every 15 min with contrast phase using Lux2 (Cytosmart) for 1day 23h 0 min. The spheroid structure is similar to that of untreated HCT116-DoxR spheroid (Movie S4) until 1day 6h, when it is observed an increased detachment of cells.

Movie S14: Time course of the aspect of HCT116 resistant to doxorubicin (HCT116-DoxR) spheroid incubated for 6h with 8 μM doxorubicin followed by irradiation for 1 min with 532 nm laser. Images were acquired every 15 min with contrast phase and red fluorescence using Lux2 (Cytosmart) for 2days 0h 15min. Spheroid structure is similar to the untreated HCT116-DoxR spheroid (Movie S4). An increased cell detachment is observed after 10h, consistent with spheroid disintegration.

Movie S15: Time course of the aspect of HCT116 resistant to doxorubicin (HCT116-DoxR) spheroid incubated for 24h with 8 nM AuNP@PEG, washed three times with phosphate buffer saline, followed by irradiation for 1 min with 532 nm laser and incubation with 8 μM doxorubicin. Images were acquired every 15 min with contrast phase using Lux2 (Cytosmart) for 1day 23h 40min. Total Dox coverage of the spheroid occur after 19h incubation. After 6h, it is observed the appearance of smaller round cells at the periphery of the spheroid (lower right) that increase in number with time. Such observations are consistent with decreased spheroid integrity.

Movie S16: Time course of the aspect of spheroids of HCT116 resistant to doxorubicin (HCT116-DoxR) incubated with 8 nM AuNP@PEG, washed three times with phosphate buffer saline, followed by 6h incubation with 8 μM doxorubicin and irradiation for 1 min with 532 nm laser. Images were acquired every 15 min with contrast phase using Lux2 (Cytosmart) for 2days. The spheroid size increases after irradiation, being observed occasional cell bursting at the periphery. The detachment of cells, consistent with loss of integrity, is observed after 3h.

Movie S17: HCT116 spheroid formation over time. Images were acquired every 15 min with contrast phase using Lux2 (Cytosmart) for 3 days 21h 4min. Despite a typical spheroid

structure with round edges being observed after 2 days, it only reaches the desired 700 nm after 7 days (Figure S10).

Movie S18: HCT116 resistant to doxorubicin (HCT116-DoxR) spheroid formation over time. Images were acquired every 15 min with contrast phase using Lux2 (Cytosmart) for 3 days 17h 45min. After 2 days inoculation, it is formed a spheroid structure with visible spheroid cells in the periphery that persist throughout the time of observation.

Movies analysis

When analyzing the movies of spheroid growth and destruction, it is important to consider that spheroids are 3-dimensional structures that float at the bottom of a U-shaped well. This makes it hard for the apparatus to maintain correct focus of the spheroid. Also, a compromise needs to be made ab initio considering the most probable direction of cell growth, which sometimes does not allow to keep the spheroid centre in the image frame.

Because of this 3D optical distortion, there is an apparent difference to the observed fluorescence intensity of Dox between Figures acquired in the Ti-U Eclipse inverted microscope and Movies acquired using Lux2. As an example, while the fluorescence attributed to Dox in HCT116 spheroids is detected on the periphery after 1h30min (Figure 3A), an increased fluorescence is observed in the middle of the spheroid only after 4 h in Movie S7. This occurs due to the different focal points and fluorescence acquisition settings used to visualize the spheroids, which are a limitation of the equipment. In the real-time analysis, the spheroid surface is focused and imaged, while in the time-point images obtained in the microscope, the focus frame was targeting the center of the spheroid.

On the other side, the settings for acquisition of the red fluorescence in Lux2 could only be predicted when starting the experiment and subsequently adjusted in the software. Even though the same settings were used for all the movies, the fluctuating spheroid makes it difficult to correct fluorescence. Hence, direct (video) observation of fluorescence in the real-time analysis should not be considered for drug internalization, and cannot be used for comparison between movies and figures and between different movies since there are no procedures for quantitative correction and/or calibration of these.

International Journal of
Molecular Sciences

MDPI

Supplementary Figures:

Note: When analyzing the images with CellTox and Dox fluorescence, it should be noted that DMSO (Dox vehicle) affects the background fluorescence of the CellTox green dye in the red filter (used also to analyze the fluorescence of Dox, Figure S2). As such, analysis of fluorescence attributed to Dox in the presence of CellTox must consider this effect. Hence, figures of spheroids incubated with Dox presented in the manuscript correspond to spheroids only incubated with Dox, without CellTox dye.

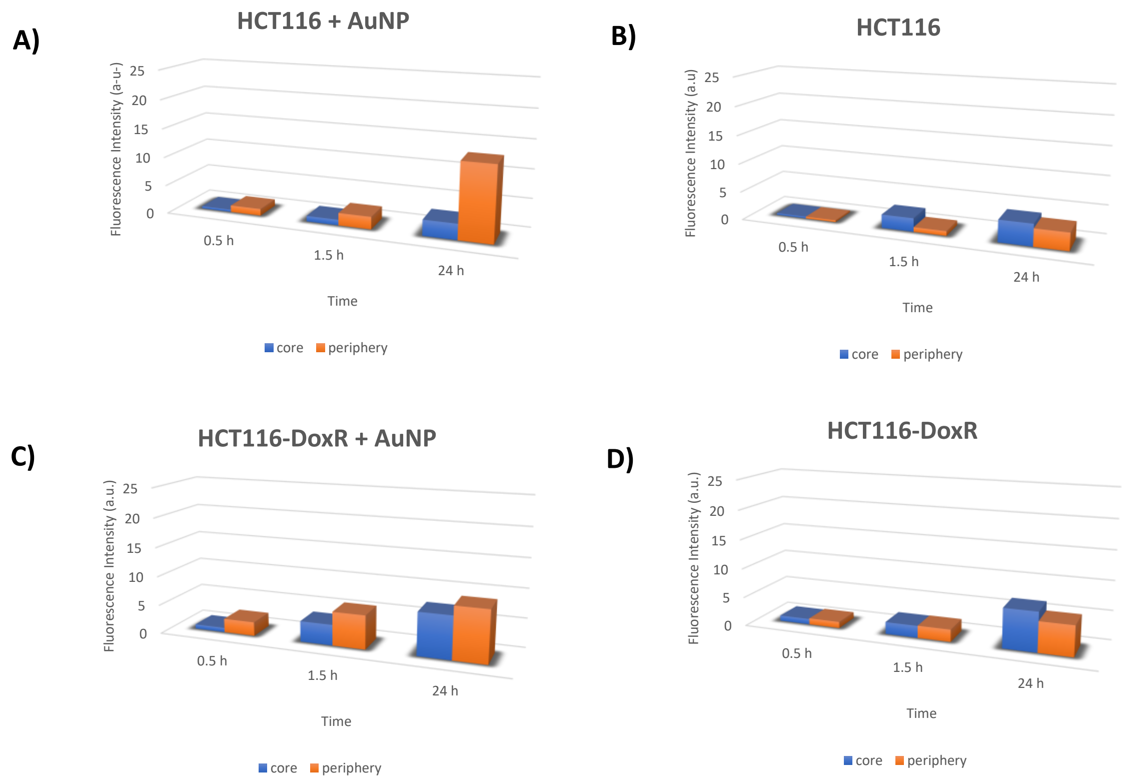

Figure S1. Variation of CellTox fluorescence in HCT116 and HCT116-DoxR spheroids incubated with and without AuNP@PEG with time. Bars represent the averaged integrated fluorescence intensity of images of three different z-stacks of the peripheral and core zone (corresponding to middle layer and innermost core) of the spheroid, acquired with a green fluorescence filter.

International Journal of *Molecular Sciences*

MDPI

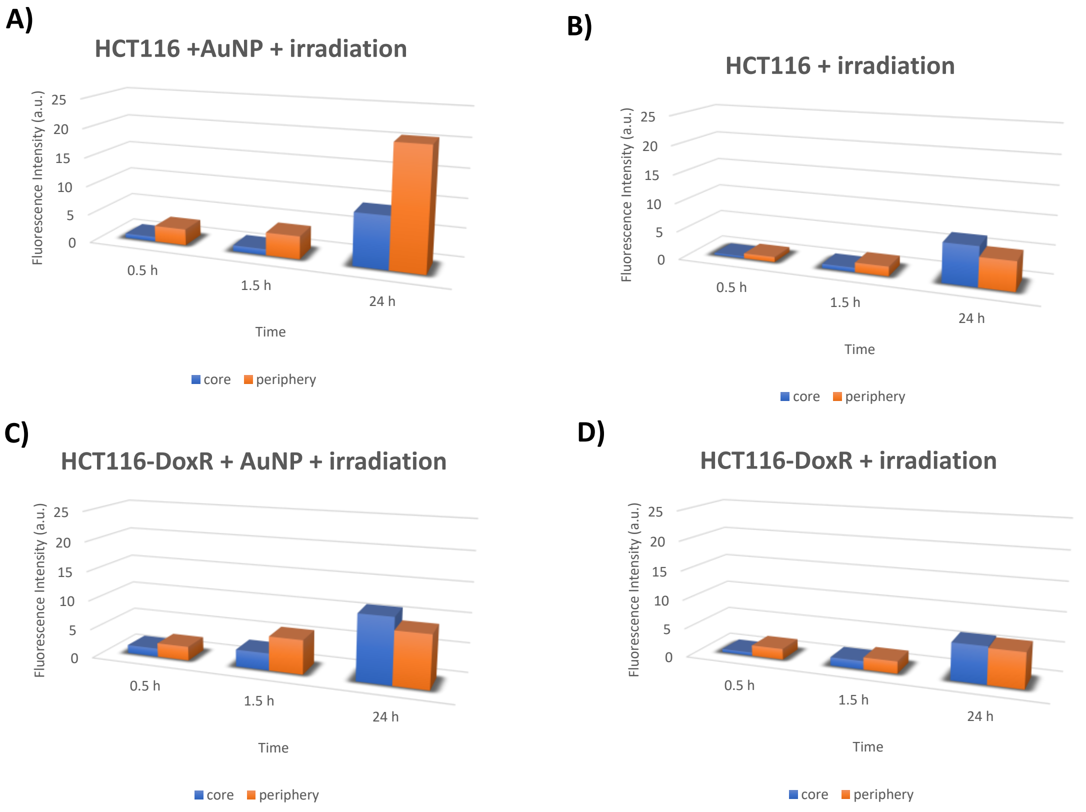

Figure S2. Variation of CellTox **fl**uorescence in irradiated HCT116 and HCT116-DoxR spheroids incubated with and without AuNP@PEG with time. Bars represent the averaged integrated fluorescence intensity of images of three different z-stacks of the peripheral and core zone (corresponding to middle layer and innermost core) of the spheroid, acquired with a green fluorescence filter.

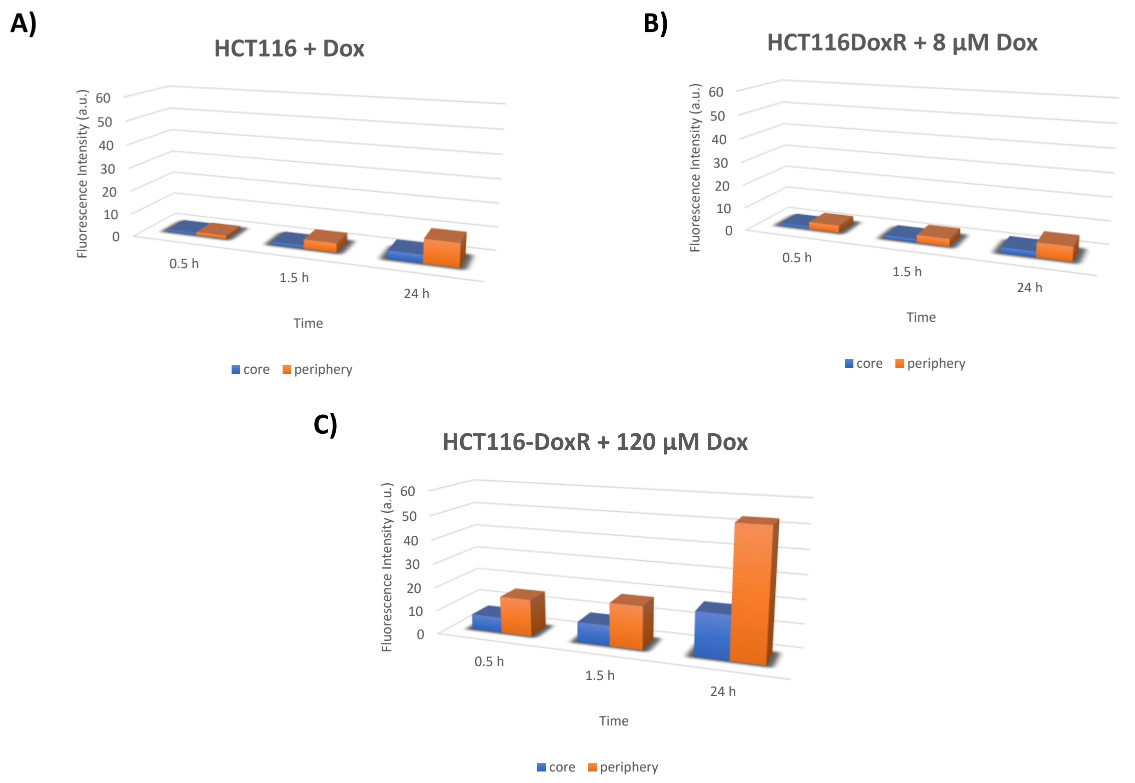

Figure S3. Variation of Dox fluorescence in HCT116 and HCT116-DoxR spheroids with time. Bars represent the averaged integrated fluorescence intensity of images of three different z-stacks of the peripheral and core zone (corresponding to middle layer and innermost core) of the spheroid, acquired with a red fluorescence filter.

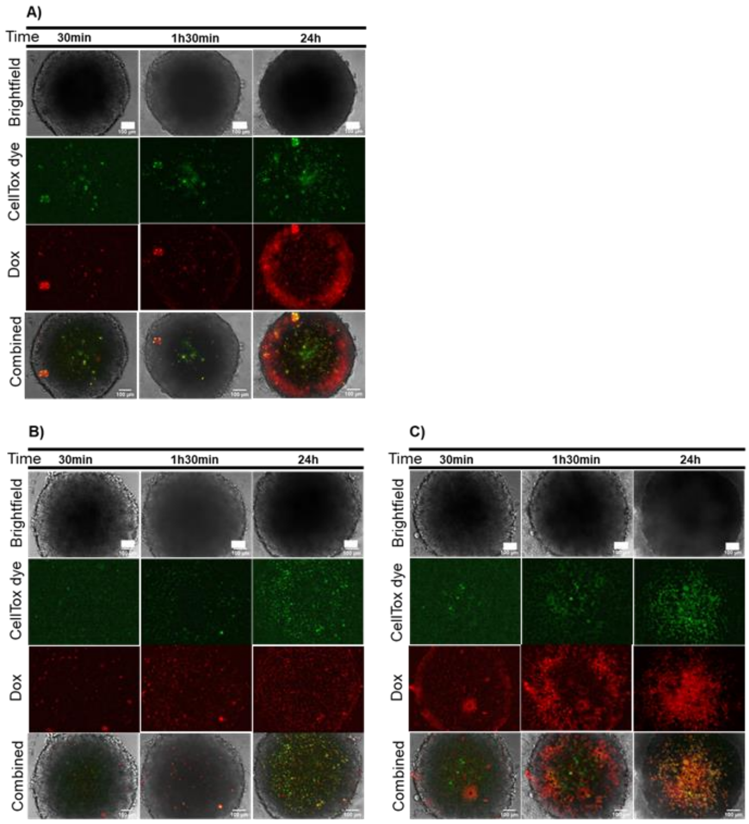

Figure S4. Effect of Doxorubicin (Dox) on cell viability measured by CellTox Dye. A) HCT116 spheroids incubated with 8 μM Dox; B) Doxorubicin resistant HCT116 (HCT116-DoxR) spheroids incubated with 8 μM Dox; C) HCT116-DoxR spheroids incubated with 120 μM Dox. Microscopy images were acquired in Brightfield, with a green fluorescence filter (to evaluate CellTox green dye), or with a red fluorescence filter (to evaluate Dox penetration) after 30 min, 1h30min or 24h incubation with CellTox dye. The combined image result from the overlap between brightfield, green filter and red filter images. Scale bar corresponds to 100 μm.

Figure S4 shows that incubation of different HCT116 spheroids with Dox retrieved different outcomes. In Dox sensitive HCT116 spheroids, the accumulation of the drug, visualized by an increased red fluorescence intensity, is only observed after 24h at the spheroid outer layer (Figure S4A). Cell viability remains similar throughout time with an increased intensity, associated with cell death, at core of the spheroid. Exposure of HCT116-DoxR spheroids to the Dox concentration used to expose HCT116 sensitive spheroids, showed no Dox accumulation and decreased cell viability after 24h, (Figure S4B). However, after exposure to higher Dox concentration, 120 μM, the drug accumulation is observed in the periphery after 30 min, in the

middle area after 1h30min and at the core after 24h (Figure S4C). No alterations on cell viability were detected during the time course of the experiment. Importantly, the cell viability of HCT116 and HCT116-DoxR spheroids incubated with 8 μM Dox is like untreated spheroids (Figure 4B and 4D, respectively), and with spheroids incubated with Dox vehicle, DMSO (Figure S5).

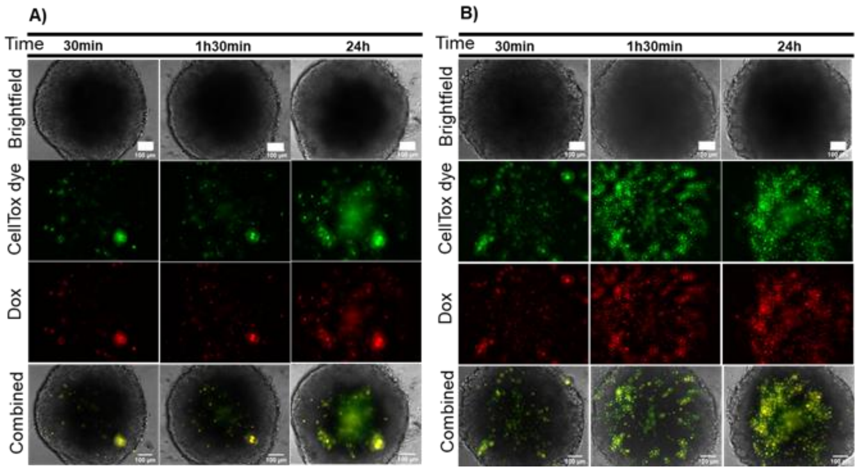

Figure S5. Cell viability of HCT116 spheroids in the presence of DMSO evaluated by CellTox green dye. A) HCT116 spheroids incubated with 0.1 % (v/v) DMSO; B) Doxorubicin resistant HCT116 (HCT116-DoxR) spheroids incubated with 0.1 % (v/v) DMSO. Microscopy images were acquired in Brightfield, with a green fluorescence filter (to evaluate CellTox green dye), or with a red fluorescence filter (to evaluate leakage of fluorescence in red filter) after 30 min, 1h30min or 24h incubation with CellTox dye. The combined image result from the overlap between brightfield, green filter and red filter images. Scale bar corresponds to 100 μm.

For comparison with results of spheroids exposure to Dox, it was analyzed the red fluorescence with the same exposure settings applied to Dox fluorescence analysis. Red fluorescence was also observed in the spheroids. However, the composite image showed that fluorescence in red filter is co-localized with high intensity green fluorescence, suggesting that both are associated with the CellTox fluorescence. The cell viability of HCT116 spheroids incubated with DMSO, the Dox vehicle, was similar in the first 1h30min of incubation, but after 24h exposure the cell viability in the core of spheroid decreased. On the other side, it is observed a decreased cell viability in the middle and center of HCT116-DoxR spheroids, right after 1h30min incubation with DMSO that remained similar until at least 24h.

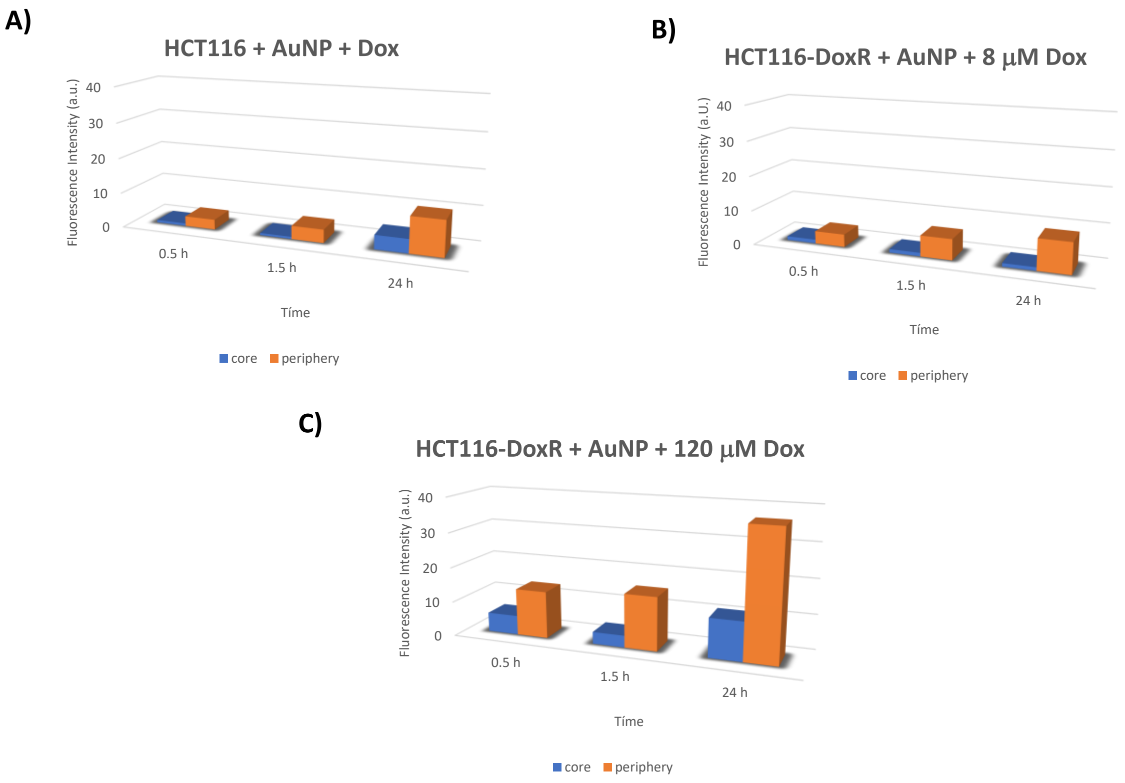

Figure S6. Variation of Dox fluorescence in HCT116 and HCT116-DoxR spheroids incubated with AuNP@PEG and Dox with time. Bars represent the averaged integrated fluorescence intensity of images of three different z-stacks of the peripheral and core zone (corresponding to middle layer and innermost core) of the spheroid, acquired with a red fluorescence filter.

International Journal of *Molecular Sciences*

MDPI

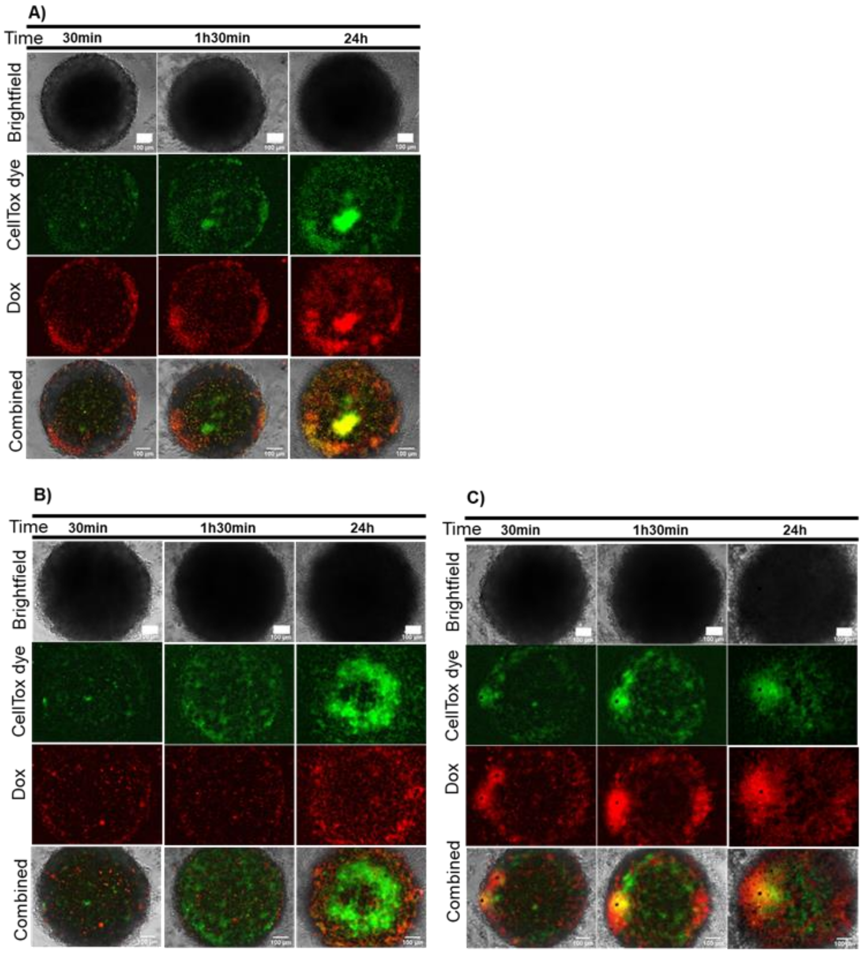

Figure S7. Cell viability of HCT116 spheroids after incubation with AuNPs and Doxorubicin (Dox) evaluated by CellTox green dye. A) HCT116 spheroids incubated with 8 nM AuNP@PEG, followed by incubation with 8 µM Dox; B) Doxorubicin resistant HCT116 (HCT116-DoxR) spheroids incubated with 8 nM AuNP@PEG, followed by incubation with 8 µM Dox; C) HCT116-DoxR spheroids incubated with 8 nM AuNP@PEG, followed by incubation with 120 µM Dox. Microscopy images were acquired in Brightfield, with a green fluorescence filter (to evaluate CellTox green dye), or with a red fluorescence filter (to evaluate Dox penetration) after 30 min, 1h30min or 24h incubation with CellTox dye. The combined image result from the overlap between brightfield, green filter and red filter images. Scale bar corresponds to 100 µm.

The pre-incubation of HCT116 spheroids with AuNP@PEG followed by Dox, resulted in a decreased cell viability in the spheroid periphery after 1h30 and a general loss of viability in the spheroid after 24h, with emphasis in the intermediary core (Figure S7A). Regarding HCT116-DoxR pre-incubated with AuNP@PEG followed by 8 µM Dox, it is observed a general decreased cell viability throughout the spheroid after 1h30 and a high number of dead

cells in the intermediary core after 24h (Figure S7B). On the other side, the exposure to 120 μM Dox after AuNP@PEG incubation, induce cell death at the spheroid periphery right after 30 min incubation, that spreads to the entire spheroid after 24h (Figure S7C).

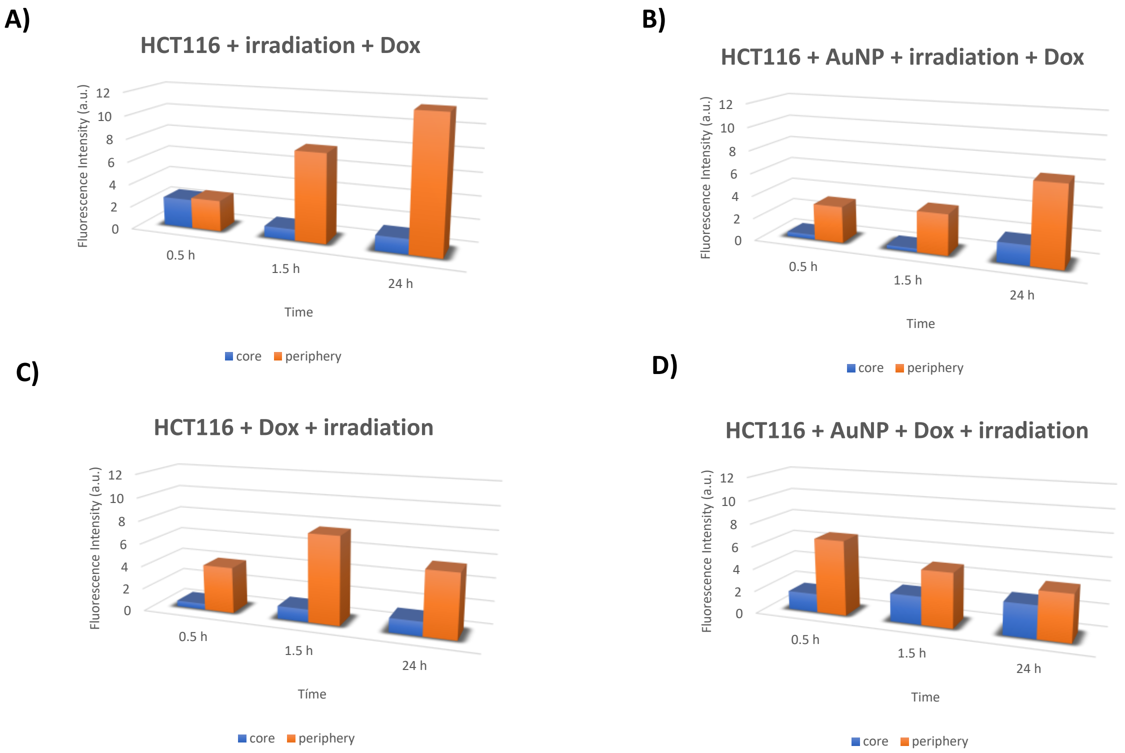

Figure S8. Variation of Dox fluorescence in irradiated HCT116 spheroids incubated with and without AuNP@PEG with time. Bars represent the averaged integrated fluorescence intensity of images of three different z-stacks of the peripheral and core zone (corresponding to middle layer and innermost core) of the spheroid, acquired with a red fluorescence filter.

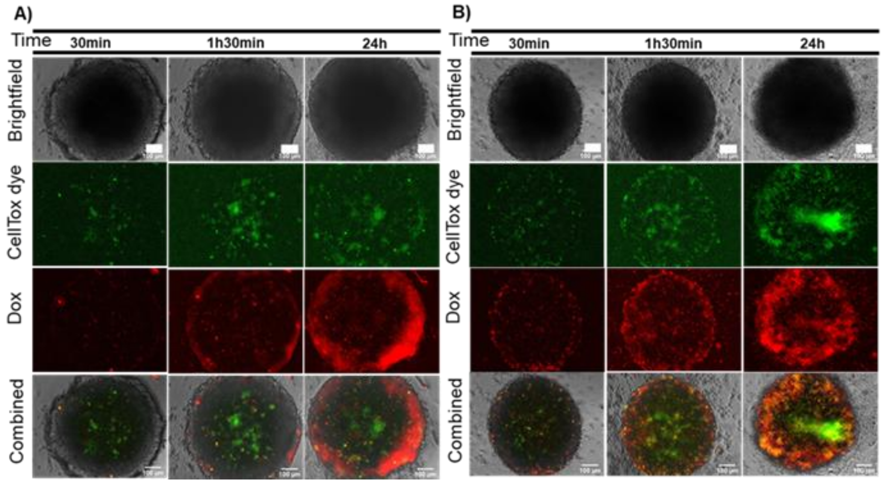

Figure S9. AuNP@PEG and irradiation on Doxorubicin (Dox) penetration and HCT116 spheroids cell viability evaluated by CellTox green dye. A) HCT116 spheroids irradiated for 1 min with 532 nm laser, and then incubated with 8 μM Dox; B) HCT116 spheroids incubated with 8 nM AuNP@PEG, irradiated for 1 min with 532 nm laser and then incubated with 8 μM Dox. Microscopy images were acquired in Brightfield, with a green fluorescence filter (to evaluate CellTox green dye), or with a red fluorescence filter (to evaluate Dox penetration) after 30 min, 1h30min or 24h incubation with CellTox dye. The combined image result from the overlap between brightfield, green filter and red filter images. Scale bar corresponds to 100 μm.

The irradiation followed by Dox exposure of HCT116 spheroids caused an increased cell death at the core of spheroid after 1h30min that was spread to all the spheroid after 24h (Figure S9A). A pre-incubation with AuNP@PEG induced earlier and increased cell death in all the spheroid with particular emphasis in spheroid periphery after 1h30min and in intermediary section after 24h (Figure S9B). These results showed that AuNP@PEG followed by irradiation improved the therapeutic effect of Dox.

International Journal of
Molecular Sciences

MDPI

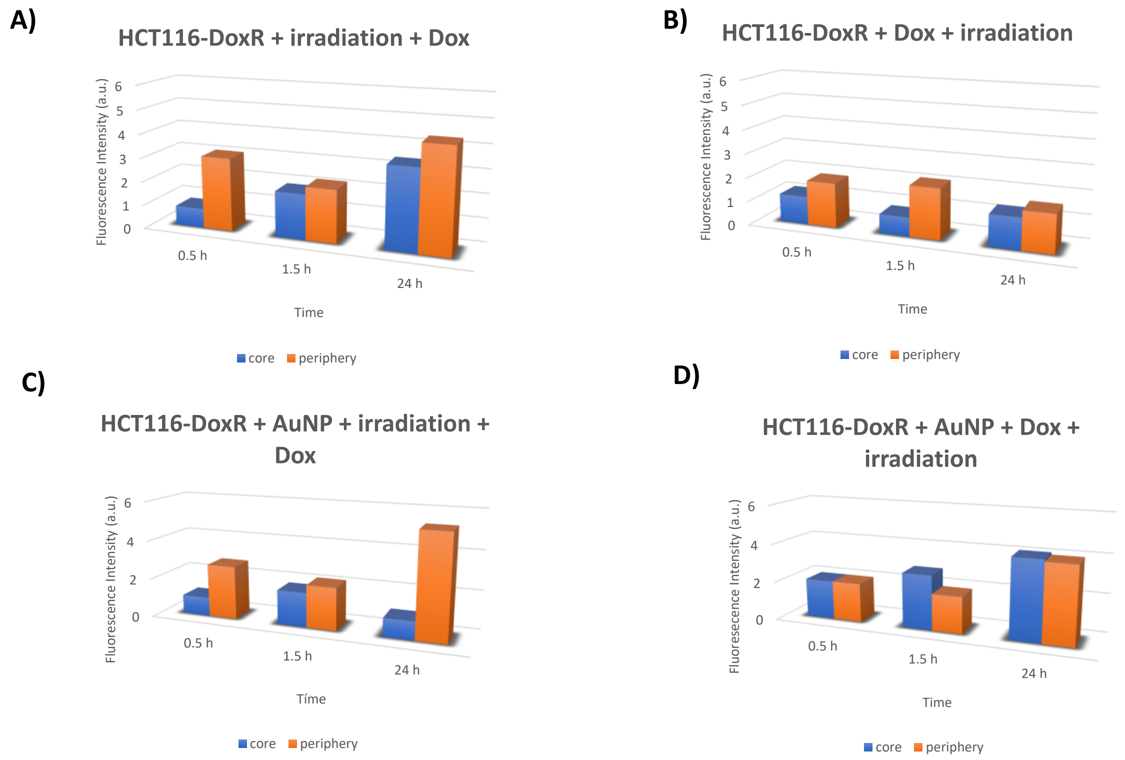

Figure S10. Variation of Dox fluorescence in irradiated HCT116-DoxR spheroids incubated with and without AuNP@PEG with time. Bars represent the averaged integrated fluorescence intensity of images of three different z-stacks of the peripheral and core zone (corresponding to middle layer and innermost core) of the spheroid, acquired with a red fluorescence filter.

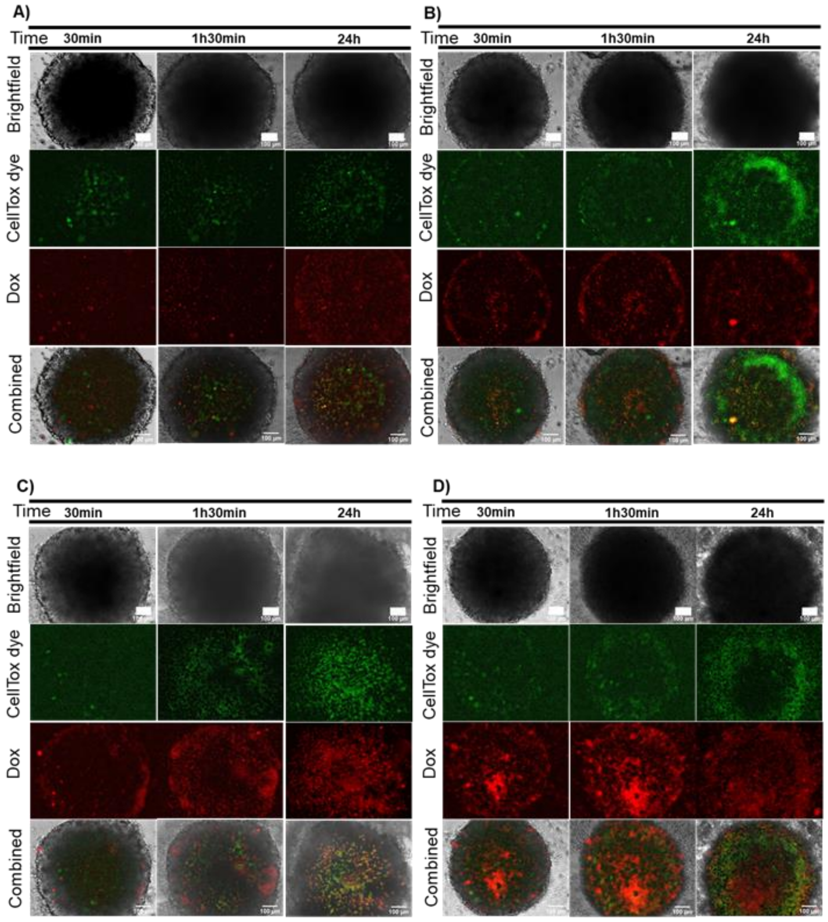

Figure S11. AuNP@PEG and irradiation effect in Doxorubicin (Dox) penetration and cell viability evaluated by CellTox green dye. A) HCT116-DoxR spheroids irradiated for 1 min with 532 nm laser, and then incubated with 8 µM Dox; B) HCT116-DoxR spheroids incubated with 8 nM AuNP@PEG, irradiated for 1 min with 532 nm laser, and then incubated with 8 µM Dox; C) HCT116-DoxR spheroids irradiated for 1 min with 532 nm laser, and then incubated with 120 µM Dox; D) HCT116-DoxR spheroids incubated with 8 nM AuNP@PEG, irradiated for 1 min with 532 nm laser, and then incubated with 120 µM Dox. Microscopy images were acquired in Brightfield, with a green fluorescence filter (to evaluate CellTox green dye), or with a red fluorescence filter (to evaluate Dox penetration) after 30 min, 1h30min or 24h incubation with CellTox dye. The combined image result from the overlap between brightfield, green filter and red filter images. Scale bar corresponds to 100 µm.

The irradiation followed by 8 µM Dox incubation of HCT116-DoxR spheroid showed a similar cell viability throughout time (Figure S11A). The pre-exposure to AuNP@PEG showed a different outcome, decreasing cell viability at the periphery of the spheroid after 30 min, and

International Journal of
Molecular Sciences

MDPI

increasing cell death in the intermediary region after 24h (Figure S11B), an effect also observed in AuNP@PEG exposure followed by incubation with 8 μM Dox (Figure S7B). On the other hand, irradiation followed by exposure to higher concentration of the drug, 120 μM Dox, induced a progressive increased cell death (Figure S11C). Interestingly, similarly to the response to the lower Dox concentration (Figure S11B), AuNP@PEG pre-incubation, also induced progressive increased cell death but in the intermediary region of the spheroid (Figure S11D).

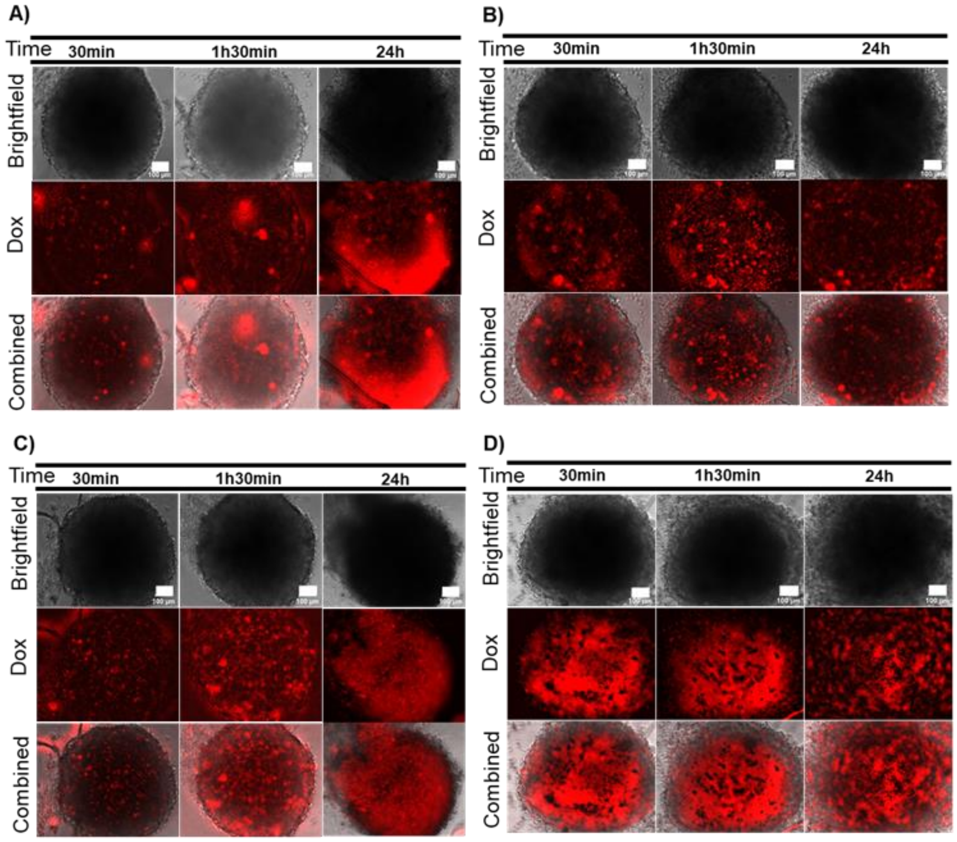

Figure S12. Effect of AuNP@PEG and irradiation in doxorubicin (Dox) diffusion in Dox resistant HCT116 (HCT116-DoxR) spheroids. A) HCT116-DoxR spheroids irradiated with 532 nm laser for 1 min and then incubated with 120 μM Dox; B) HCT116-DoxR spheroids incubated for 6 h with 120 μM Dox and then irradiated with 532 nm laser for 1 min; C) HCT116-DoxR spheroids incubated with 8 nM AuNP@PEG, irradiated with 532 nm laser for 1 min and then incubated with 120 μM Dox; D) HCT116-DoxR spheroids incubated with 8 nM AuNPs, incubated for 6 h with 120 μM Dox and then irradiated with 532 nm laser for 1 min. Microscopy images were acquired in Brightfield or with a red fluorescence filter after 30

min, 1h30min or 24h incubation. The combined image result from the overlap between brightfield and red filter images. Scale bar corresponds to 100 µm.

In the pre-irradiated HCT116-DoxR spheroids, an increased Dox penetration was only observed after 24h with accumulation mainly in the periphery and intermediary region (Figure S12A). On the other side, post-irradiation allowed the immediate entry of the drug with higher accumulation at the spheroid periphery (Figure S12B). The incubation with AuNP@PEG previous to the strategies referred above showed an induced Dox penetration and spheroid degradation. The pre-treatment with AuNP@PEG and irradiation induced Dox penetration, with increased red fluorescence throughout the spheroid after 1h30min and accumulation in the intermediary region after 24h, with compromised spheroid integrity (Figure S12C). As for the HCT116-DoxR spheroid incubation with lower Dox concentration (Figure S12D), pre-incubation with AuNP@PEG, followed by Dox and subsequent irradiation induced Dox penetration right after 30 min, with subsequent spheroid disintegration (Figure S12D).

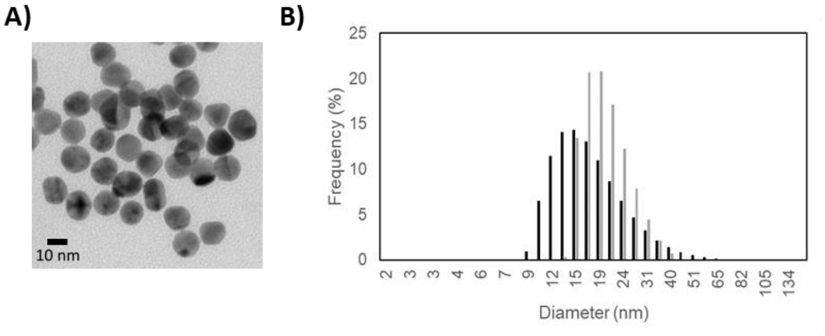

C)

Particle	*Size (nm)*	*Zeta potential (mV)*
AuNP	14.3 ± 0.5	- 37.4 ± 6.7
AuNP@PEG	18.4 ± 0.3	-13.3 ± 2.2

Figure S13. Functionalization of AuNP@PEG. A) TEM image of citrate caped AuNPs. B) Dynamic light scattering with diameter distribution of AuNPs (black bars) and AuNP@PEG

(grey bars). C) Size and zeta potential of citrate caped AuNPs and AuNP@PEG. The increased hydrodynamic diameter of AuNP@PEG relative to citrate caped AuNPs suggest a successful functionalization with PEG.

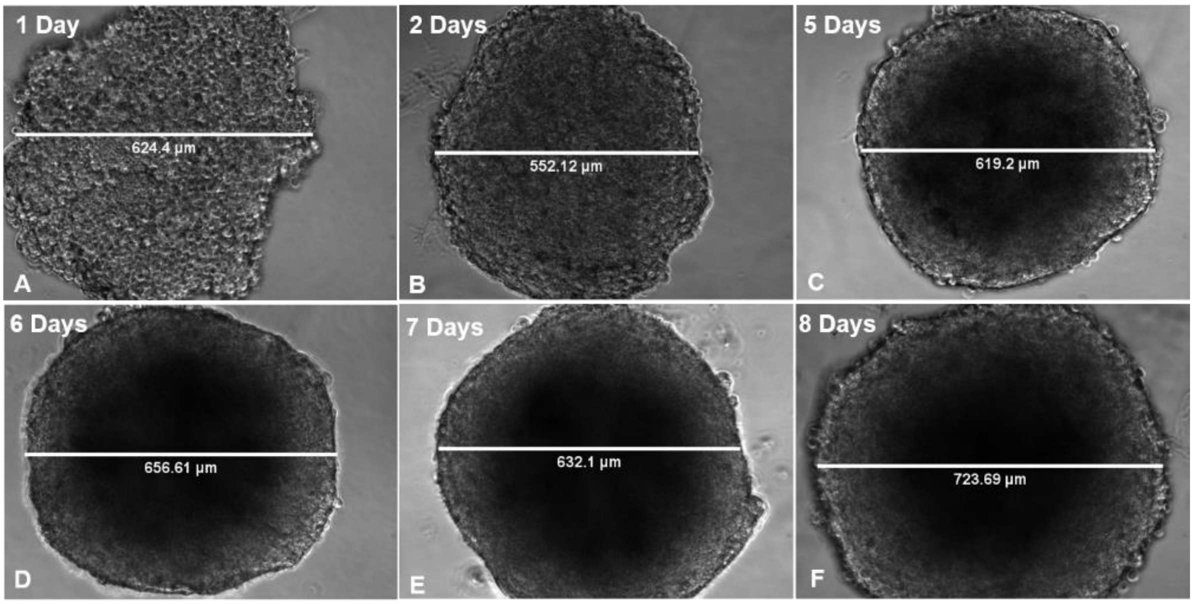

Figure S14. Diameter size and aspect of HCT116 spheroids formation throughout time. (A) 1 day with 624.4 µm (B) 2 days with 552.1 µm of diameter (C) 5 days with 619.2 µm (D) 6 days with 656.61 µm (E) 7 days with 632.1 µm (F) 8 days with 723.69 µm of diameter. Images were acquired in phase contrast with 200x amplification. Lines in white were used to measure the spheroid diameter in ImageJ software.
